# Optimization of Spent Coffee Grounds as Fat and Flour Substitutes in Gluten‐Free Cakes: Effects on Quality Characteristics

**DOI:** 10.1002/fsn3.72029

**Published:** 2026-06-15

**Authors:** Gizem Tiryaki, Emine Nakilcioğlu

**Affiliations:** ^1^ Food Engineering Department, Engineering Faculty Ege University Izmir Türkiye

**Keywords:** food waste, gluten‐free cake, spent coffee grounds, substitute, waste valorization

## Abstract

With the increasing global population and the depletion of natural resources, there is a growing interest in alternative food sources with beneficial and functional properties. Spent coffee grounds (SCGs) stand out among food waste due to their rich nutritional composition and potential for evaluation in terms of a sustainability perspective. In this study, the objective was to determine the optimum utilization level of SCGs as a rice flour and fat substitute in gluten‐free cake formulations. Response surface methodology (RSM) was employed to determine the optimum formulation to produce gluten‐free cakes with SCG substitute. The effects of SCG substitution on fat content (0%, 20%, 40% [w/w]) and rice flour content (0%, 25%, 50% [w/w]) were investigated in terms of chemical, physical, and sensory properties of gluten‐free cakes. The addition of SCG was found to significantly influence the substitution levels of fat and rice flour (*p* < 0.05). The optimum values for the independent variables were found as 36.75% fat substitute and 12.38% rice flour substitute. The optimum formulation confirmed the adequacy and predictive capability of regression models. In the optimum formulation of gluten‐free cake substituted with SCGs, the baking loss was found to be 9.37%, L*_crust_ and b*_crust_ values were 43.87 and 8.06, both springiness and cohesiveness values were 0.18, total phenolic content was 1.11 mg GAE/g, antioxidant activity value was 1.59 mg TEAC/g for DPPH assay and 0.78 mg TEAC/g for ABTS assay, and overall acceptability was 6.75. Also, the addition of SCG resulted in approximately a 7‐fold increase in dietary fiber content (from 2.3 g/100 g to 15.71 g/100 g). It is anticipated that gluten‐free cakes formulated with SCGs may serve as an alternative product for individuals with gluten sensitivity and raise awareness about the valorization of food waste. The recovery of food by‐products and the development of functional food products are expected to play a key role in the sustainable food technologies of the future.

## Graphical abstract

This study aimed to develop gluten‐free cakes with spent coffee grounds as fat and flour substitutes using RSM. The optimum values for the use of spent coffee grounds were found to be 36.75% fat substitute and 12.38% rice flour substitute. In the optimum formulation of gluten‐free cake substituted with SCGs, the baking loss was determined as 9.37%, L*_crust_ and b*_crust_ values as 43.87 and 8.06, both springiness and cohesiveness values as 0.18, total polyphenols as 1.11 mg GAE/g, antioxidant activity as 1.59 mg TEAC/g for DPPH assay and 0.78 mg TEAC/g for ABTS assay, and overall acceptability as 6.75.

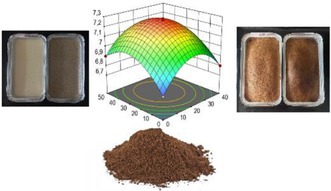

## 1 Introduction

Coffee (*Coffea* sp.) is one of the most consumed beverages worldwide due to its distinctive aroma and taste and has a leading position in the hot beverage market (Mahmud et al. 2020; Šeremet et al. 2022). The prevalence of coffee consumption generates a significant amount of organic waste, particularly spent coffee grounds (SCGs), throughout the production and consumption processes. The generation of approximately 0.91 g of SCG per gram of coffee is a situation that should be taken into consideration in terms of environmental sustainability (Mussatto et al. 2011; Sulyman et al. 2020). Without effective waste management, food loss is expected to reach 2.1 billion tons and economic losses to reach $1.5 trillion by 2030 (Saberian et al. 2021).

SCG, obtained by grinding and brewing coffee beans, attracts attention with its rich nutritional content in terms of both functional components and energy value (Wang et al. 2022; Zhao et al. 2024). SCG contains approximately 50%–60% carbohydrates, 17% protein, 7%–20% lipids, and 1%–2% ash. It is also a rich source of vitamins B and E, potassium (3–11 mg/g), magnesium (2 mg/g), and calcium (1.2 mg/g) (Ballesteros et al. 2014; Procida et al. 2020; Bomfim et al. 2022). Additionally, SCG is a food waste containing 12 mg/g phenolic compounds and a relatively low amount of caffeine (14.5 μg/g) (Bomfim et al. 2022).

Due to its high dietary fiber, protein, lipid, and polyphenol content, SCG has been evaluated as a functional ingredient in food formulations in recent years (Choe 2025). SCG has a high dietary fiber content ranging from 50% to 65% (Trà et al. 2021; Romauli et al. 2023; Papageorgiou et al. 2024). Soluble fiber constitutes 20% of this dietary fiber content, whereas the remaining 80% consists of insoluble fiber (Ballesteros et al. 2014). The high insoluble dietary fiber content of SCG positively affects the intestinal flora by supporting digestive health (Montemurro et al. 2024). The dietary fibers obtained from SCG are considered antioxidant dietary fibers that may also benefit against oxidative stress and can be used as a functional food ingredient (Papageorgiou et al. 2024). Thanks to its high dietary fiber content, SCG's water and fat‐binding capacities ensure emulsion stability in foods, thereby stabilizing high‐fat products and reducing fat absorption through its soluble fibers. This also influences sensory properties. The ability of soluble fibers to reduce fat absorption offers excellent potential for using SCG as a raw material in the development of functional foods (Ballesteros et al. 2014). Thanks to the water‐binding capacity of dietary fibers, free water is immobilized in the product matrix, which contributes to maintaining product stability even in the absence of fat (Schmiele et al. 2015; Papageorgiou et al. 2024). In literature, it has been reported that coffee by‐products can be used as fat replacers in cake formulations and allow for fat reduction up to certain levels without causing a significant loss in product quality (Ateş and Elmacı 2019).

Furthermore, SCG's particle structure and fiber content also exhibit significant functional effects in terms of flour substitution (Ahanchi et al. 2024). This fiber‐rich structure directly influences the product's volume, pore structure, and textural properties by altering water distribution and rheological properties in dough systems (Hussein et al. 2019; Jo et al. 2026). Particularly in gluten‐free systems, fiber sources have been reported to contribute to matrix formation due to the weak structural network. However, an increased fiber content may limit gas retention capacity, leading to a reduction in volume and an increase in hardness (Kiumarsi et al. 2019; Bender and Schönlechner 2020).

Furthermore, SCG is abundant in polyphenolic compounds such as chlorogenic acid (up to 2.20 mg/g) and caffeic acid (up to 2.22 mg/g), which exhibit protective effects against cancer, obesity, liver disorders, and cardiovascular diseases (Montemurro et al. 2024; Castaldo et al. 2021; Mussatto et al. 2011; Ballesteros et al. 2014). Due to its rich nutritional content and positive health effects, SCG stands out as a food ingredient suitable for use in the production of value‐added products (Ahmed et al. 2024).

In line with sustainability goals, the reuse of food waste is becoming increasingly important. SCG, an organic waste with a rich nutritional composition, has significant potential for both health and sustainable food production (Saratale et al. 2020; Papageorgiou et al. 2024). Alternative applications of SCG include its use as a fertilizer in plant cultivation (Chrysargyris et al. 2021; Horgan et al. 2023; Flores et al. 2024), and as an animal feed supplement (de Otálora et al. 2020; Angeloni et al. 2024). SCG is also used in the production of fuels such as biogas and biodiesel (Changotra et al. 2024; Alshuaib et al. 2025; Saxena et al. 2025). SCG also has a wide range of applications in the food sector (Yusufoğlu et al. 2024). The use of SCG in functional food production not only increases the nutritional value of the final product but also reduces environmental waste. Thanks to its high dietary fiber and antioxidant content, SCG can be used as a substitute for certain ingredients in food products (Ballesteros et al. 2014; Campos‐Vega et al. 2015; Franca and Oliveira 2022).

Gluten is widely preferred in many food products due to its functional properties (Zhang et al. 2023). However, gluten consumption can cause immune‐related health problems, such as celiac disease (0.35%–1%) and other gluten‐related disorders (1%–3%), in some individuals (Arámburo‐Gálvez et al. 2020; Sabença et al. 2021). The solution to these health problems is possible by implementing a strict gluten‐free diet program (Mazzola et al. 2024). Today, a gluten‐free diet is also preferred by individuals who choose a healthy lifestyle and avoid gluten consumption for reasons such as weight management and digestive problems (Arslain et al. 2021). However, the increasing demand for gluten‐free products, limited gluten‐free food options, and high costs make it difficult to adhere to such diets (Wieser et al. 2021; Demirkesen and Ozkaya 2022). Therefore, it is crucial to develop new products to offer alternative foods for individuals on a gluten‐free diet and to prevent nutritional deficiencies in these individuals.

Nowadays, there is an increasing consumer demand for products with reduced calories and fat content and enriched with dietary fiber and bioactive ingredients. Popular snacks such as cakes are typically characterized by high levels of calories, fat, and flour. Therefore, the use of certain dietary fiber and bioactive compound sources as fat and flour substitutes has become increasingly common to offer healthier alternatives (Oseguera‐Castro et al. 2019; Campos‐Vega et al. 2020; Severini et al. 2020). The studies in literature indicate that SCG has been utilized as a wheat flour substitute in various bakery products including sponge cakes, biscuits, and cookies (Vázquez‐Sánchez et al. 2018; Hussein et al. 2019; Desai et al. 2020; Oliveira Batista et al. 2023). To our knowledge, SCG has not been used as fat and rice flour substitute in gluten‐free cake formulation, and its usage rates have not been optimized. Fat substitution is particularly important due to growing consumer demand for low‐fat baked goods, whereas rice flour substitution is noteworthy for its potential to increase the dietary fiber content and improve the nutritional value of the product. However, such substitutions can significantly affect fundamental quality characteristics such as texture, cake volume, color, and sensory acceptability. The main objective of this study is to determine the optimum usage ratios of SCGs as a substitute for fat and rice flour in a gluten‐free cake formulation and to maximize improvements in the physical, chemical, and sensory properties of gluten‐free cakes. Another objective is to evaluate the effects of optimum SCGs addition on the quality characteristics (physical, chemical, and sensory) of gluten‐free cakes by comparing them to a control cake and to obtain novel and healthier gluten‐free cakes containing SCGs as a functional component.

## 2 Materials and Methods

### 2.1 Materials and Chemicals

Rice flour, oil, eggs, milk, sugar, and baking powder used in gluten‐free cake production were purchased from a local market in İzmir, Turkey. SCG samples were obtained from a chain restaurant using Robusta type filter coffee (Indonesia, Java, grade 1). They were dried in a tray dryer (Eksis, Turkey) at 50°C and 1 m/s air speed until the moisture content reached constant weight (Vázquez‐Sánchez et al. 2018). The dried samples were stored in locked aluminum polyethylene packages at 4°C until the analysis. All chemicals used in the analysis were purchased from Sigma Chemical Co. (St. Louis, MO, USA).

### 2.2 Experimental Design and Optimization

Response surface methodology (RSM) was used to optimize the use of SCG as a fat and rice flour substitute in the gluten‐free cake samples. A Face‐Central Composite Design (FCCD) was constructed using Design‐Expert software (version 13.0; Stat‐Ease Inc., Minneapolis, MN, USA) to evaluate the effects of two independent variables: fat substitute (*X*_1_: 0%–40%) and rice flour substitute (*X*_2_: 0%–50%). Based on the independent variables, a total of 13 runs—including five replicates at the center point—were generated (Table 1).

**TABLE 1:** Experimental design matrix with independent and dependent variables of FCCD.

Run	Independent variables		Dependent variables									
	*X*_1_	*X*_2_	*R*_1_	*R*_2_	*R*_3_	*R*_4_	*R*_5_	*R*_6_	*R*_7_	*R*_8_	*R*_9_	*R*_10_
1	0	0	10.26	49.75	12.08	0.25	0.27	2.30	0.22	0.34	0.19	6.73
2	0	50	9.30	42.98	4.89	0.23	0.12	14.70	0.79	1.47	0.68	6.90
3	20	25	9.19	44.43	6.73	0.13	0.13	14.60	0.74	1.05	0.44	7.26
4	0	25	9.04	44.32	6.32	0.20	0.15	9.04	0.77	0.66	0.43	7.00
5	20	50	9.33	40.09	2.77	0.16	0.14	21.76	1.43	1.92	1.57	7.12
6	20	25	8.76	44.15	6.49	0.15	0.16	15.80	0.84	1.29	0.69	7.28
7	20	25	8.29	43.86	5.61	0.17	0.13	15.50	0.91	1.87	1.00	7.25
8	20	0	10.66	49.15	11.64	0.22	0.27	3.56	0.51	0.19	0.25	7.00
9	40	50	9.17	40.13	3.69	0.15	0.17	27.93	1.46	2.34	1.87	6.90
10	40	0	10.98	44.71	7.50	0.20	0.22	9.60	0.70	0.95	0.53	6.80
11	20	25	8.70	44.72	6.98	0.15	0.14	14.00	0.96	1.42	0.70	7.26
12	20	25	8.39	45.58	7.37	0.16	0.14	12.50	0.89	1.47	0.69	7.25
13	40	25	9.27	40.71	5.73	0.16	0.14	25.29	1.39	2.43	1.05	7.02

Abbreviations: FCCD, Face‐Central Composite Design; *R*_1_, baking loss (%); *R*_10_, overall acceptability; *R*_2_, L*_crust_; *R*_3_, b*_crust_; *R*_4_, springiness; *R*_5_, cohesiveness; *R*_6_, total dietary fiber content (g/100 g); *R*_7_, total phenolic content (mg GAE/g); *R*_8_, antioxidant capacity measured by DPPH method (mg TEAC/g); *R*_9_, antioxidant capacity measured by ABTS method (mg TEAC/g); *X*_1_, fat substitute (%); *X*_2_, rice flour substitute (%).

For each run, SCG‐substituted gluten‐free cakes were produced under the specified conditions and analyzed for key response variables: baking loss (*R*_1_, %), color values measured by L*_crust_ (*R*_2_) and b*_crust_ (*R*_3_), springiness (*R*_4_), cohesiveness (*R*_5_), total dietary fiber content (*R*_6_, g/100 g), total phenolic content (TPC) (*R*_7_, mg QE/g), antioxidant capacities measured by DPPH (*R*_8_, mg TEAC/g), and ABTS (*R*_9_, mg TEAC/g), and overall acceptability (*R*_10_). During the optimization process, baking loss was constrained within a specified range, and color parameters, including L*_crust_ and b*_crust_ were set to reach their target values, whereas other response variables were adjusted to be maximum.

The experimental data were fitted to the following equation (Equation 1) for each response variable. In this equation, *R*, *β*_0_, *β*_*i*_, *β*_*ii*_, *β*_*ij*_, and *X* are the response value, constant, linear, quadratic, interaction constants, and independent variables, respectively.

(1) $$R=\beta_{0}+\sum \beta_{i}X_{i}+\sum \beta_{\mathrm{ii}}X_{\mathrm{ii}}^{2}+\sum \beta_{\mathrm{ij}}X_{i}X_{j}$$

3‐D plots were generated. The statistical significance of all models and regression terms was evaluated using analysis of variance (ANOVA) at a 95% confidence level (*p* < 0.05). Model adequacy and predictive performance were evaluated based on statistical parameters including the coefficient of determination (*R* ^2^), adjusted *R* ^2^, predicted *R* ^2^, Fisher's test value (*F*‐value), and lack‐of‐fit test. Term reduction was used for non‐significant effects, taking into account that the model hierarchy was not disrupted. The production of SCG‐substituted gluten‐free cakes and their analyses were conducted in triplicate. Statistical analysis of the physical, chemical, and sensory properties of the control gluten‐free cake and the optimum gluten‐free cake with SCG substitution was performed using an independent sample *t*‐test in the SPSS 26.0 package program (IBM, USA). Significant differences were revealed at the 95% confidence level using Duncan's multiple comparison test (*p* < 0.05).

### 2.3 Gluten‐Free Cake Production

Gluten‐free cake batter was prepared using SCGs instead of fat (0%, 20%, 40%) and rice flour (0%, 25%, 50%) by modifying the method developed by Aslan Türker et al. (2023). The gluten‐free cake formulation contains 25 g rice flour, 11 g oil, 17 g eggs, 25 g sugar, 21 g milk, 1 g baking powder, and SCG in the amounts specified by RSM. In production, eggs and sugar were mixed using a household mixer (Fakir, Germany) for 5 min, followed by the addition of milk, oil, rice flour, and baking powder, and the mixture was mixed for another 5 min at high speed. The prepared cake batter was baked in a preheated oven (Arçelik, Turkey) at 180°C for 30 min and then cooled at room temperature for 1 h.

### 2.4 Physical Characteristics of Cake Samples

Baking loss in gluten‐free cakes was calculated as the ratio of the weight of the cake batter to the weight of the baked cake (Martínez‐Cervera et al. 2015).

The specific volume of gluten‐free cakes was assessed using a Volscan Profiler volume analyzer (Stable Microsystems, England). It was calculated by dividing the cake volume by the cake weight and was expressed in cm^3^/g (Dadalı 2023).

The crust color of the gluten‐free cakes was determined using a CR300 colorimeter (Konica Minolta, Japan). The crust color parameters of the samples were recorded as L* value for lightness/darkness, and b* values for yellowness (+*b*)/blueness (−*b*).

The texture profile analysis (TPA) of gluten‐free cakes was performed with a TAXT Plus Texture Analyzer (Stable Micro Systems Ltd., Godalming, UK) using a 36 mm diameter cylinder prop (P/36R) and a 5 kg load cell (Dadalı and Elmacı 2019). Samples were compressed twice to 40% of their initial height. Compression was performed at a test speed of 1 mm/s with a 5 s duration between cycles. The springiness and cohesiveness values were calculated from the TPA curves.

### 2.5 Chemical Characteristics of Cake Samples

The moisture and ash contents of gluten‐free cakes were determined gravimetrically using a hot air oven (Dedeoğlu, Turkey) at 105°C according to AOAC method 925.10 (AOAC 2005), and a muffle furnace (Heraeus, Germany) at 550°C (AOAC 2007), respectively. Their fat contents were found using the Soxhlet extraction method (AOAC 2007). Their protein contents were measured according to the Kjeldahl method, and the conversion factor used was 6.25 (AOAC 1990). The total carbohydrate contents of gluten‐free cakes were determined according to the formula (Equation 2) below (AOAC 2007). Their energy values were estimated with Atwater caloric constants (Schakel et al. 2009).

(2) $$\begin{array}{c} \%\text{Total carbohydrate content}\\ \;=100-\left(\%\text{moisture}+\%\mathrm{ash}+\%\text{protein}+\%\mathrm{fat}\right) \end{array}$$

The dietary fiber method described by AOAC 991.43 (2000) was applied to determine the total dietary fiber content of gluten‐free cakes. Samples were treated with 50 μL α‐amylase (98°C–100°C for 30 min), 100 μL protease (30 min at 60°C), and 300 μL amyloglucosidase enzymes (30 min at 60°C), respectively. To obtain insoluble dietary fiber, the residue was washed with warm water (2 × 10 mL, 70°C), 78% (v/v) ethanol (2 × 15 mL), 95% (v/v) ethanol (2 × 15 mL), and 95% (v/v) acetone (2 × 15 mL), respectively, and the resulting precipitate was dried at 105°C. To obtain soluble dietary fiber, the resulting precipitate and water washings from the IDF procedure were subjected to a precipitating step by adding 230 mL 95% (v/v) ethanol at 60°C. The precipitate was washed and dried with the same steps as the insoluble dietary fiber. After determining the ash content using the gravimetric method (AOAC 2007) and protein content using the Dumas method (ISO 16634‐1 2008) in the total precipitate, the total dietary fiber content of the samples was calculated using the following formula (Equation 3):

(3) $$X=\frac{R-A-P-B}{m}\times 100$$

where *X* is the total dietary fiber content (%), *R* is the mass of the total precipitate (mg), *P* and *A* are the masses of ash and protein contents in the sample (mg), *B* is the mass of the precipitate of the blank sample (mg), and *m* is the mass of the sample.

The Folin–Ciocalteu assay applied by Garcia‐Salas et al. (2010), with some modifications, was used to measure the TPCs of gluten‐free cakes. 80% (v/v) methanol extracts for the determination of TPCs were prepared according to the method developed by Xu and Chang (2007). Fifty microliter extract, 3.95 mL distilled water, 250 μL Folin–Ciocalteu reagent, and 750 μL Na_2_CO_3_ (7%, w/v) were mixed and kept at 25°C for 2 h. Then, an Agilent Cary 60 UV–Vis spectrophotometer (USA) was used to measure the absorbance of the samples at 765 nm. The results were expressed as gallic acid equivalents (mg GAE/g).

The modified DPPH method described by Sun et al. (2007) and Cemeroğlu (2007) was utilized for antioxidant capacity analysis. Forty‐five microliter extract was mixed with DPPH solution, incubated for 20 min in the dark, and absorbance was measured at 515 nm. The results were given as mg Trolox equivalents (mg TEAC/g).

The modified ABTS method developed by Re et al. (1999) was employed for antioxidant capacity analysis. Forty microliter extract was mixed with ABTS•^+^ solution, and absorbance was read at 734 nm (0 and 6 min). The results were stated as mg Trolox equivalents (mg TEAC/g).

### 2.6 Sensory Characteristics of Cake Samples

To evaluate color, texture, flavor, and overall acceptability of samples, SCG‐substituted gluten‐free cakes and a control cake were presented to 60 untrained panelists consisting of graduate students and academic staff from the Department of Food Engineering at Ege University (Izmir, Turkey). Panelists scored the cake on a hedonic scale from 1 (very poor) to 9 (very good). All participants were informed about the study and provided written informed consent prior to participation in the sensory evaluation. The study protocol was approved by the Ege University IRB (Approval No: E‐93780625‐700‐2268386).

## 3 Results and Discussion

### 3.1 Model Fitting

The effects of different proportions of SCGs used as fat (*X*_1_) and rice flour substitute (*X*_2_) on baking loss (*R*_1_), color parameters (L*_crust_, *R*_2_; b*_crust_, *R*_3_), texture properties (springiness, *R*_4_; cohesiveness, *R*_5_), total dietary fiber (*R*_6_), TPC (*R*_7_), antioxidant capacities (DPPH, *R*_8_; ABTS, *R*_9_), and overall acceptability (*R*_10_) of gluten‐free cakes are presented in Table 2. Table 3 presents the model equations for the effects of independent variables on response variables. The proposed models for all responses were highly significant with values ranging from *p* < 0.0001 to *p* = 0.0005 (Table 2). No significant lack of fit was detected (*p* > 0.05) (Table 2), indicating that the models were sufficient, and the experimental data were well reproducible These models were found to have high *R* ^2^ (0.813–0.997) and adjusted *R* ^2^ values (0.775–0.995) (Table 2), thus proving a strong relationship and a good fit between the independent variables and the responses (Chen et al. 2018). The independent variables were strong in explaining the respective responses. Also, the finding that Adequate precision was greater than 4 for all responses showed that the proposed models had sufficient signal values to navigate the design space (Table 2). Additionally, the effect of two independent variables on any response was visualized with 3D graphs.

**TABLE 2:** ANOVA results of the proposed models for responses.

	*R*_1_	*R*_2_	*R*_3_	*R*_4_	*R*_5_	*R*_6_	*R*_7_	*R*_8_	*R*_9_	*R*_10_
Model	7.04	91.36	72.64	0.0143	0.0268	630.57	1.35	4.79	2.61	0.4396
*X*_1_	—	22.04	6.75	0.0046	—	97.36	0.5211	1.76	0.7776	0.0013
*X*_2_	2.81	69.31	65.89	0.0029	0.0161	184.59	0.8258	3.03	1.66	0.0253
*X*_1_ *X*_2_	—	—	—	—	—	—	—	—	0.1777	0.0012
*X*_1_^2^	—	—	—	0.0013	—	201.78	—	—	—	0.1579
*X*_2_^2^	4.23	—	—	0.0031	0.0107	276.06	—	—	—	0.0988
Lack of fit	0.6382	11.85	10.24	0.0008	0.0028	38.97	0.1595	0.7404	0.1304	0.0005
*R* ^2^	0.8611	0.8706	0.8585	0.8991	0.8839	0.9321	0.8786	0.8131	0.9017	0.9975
Adjusted *R* ^2^	0.8333	0.8448	0.8302	0.8487	0.8607	0.8982	0.8543	0.7757	0.8689	0.9957
Predicted *R* ^2^	0.7814	0.7584	0.6980	0.7080	0.7614	0.7485	0.7567	0.6779	0.7992	0.9891
CV%	3.61	2.64	16.20	7.67	10.83	16.72	15.20	24.69	22.77	0.1775
Standard deviation	0.3370	1.17	1.09	0.0142	0.0187	2.40	0.1364	0.3319	0.1778	0.0125
Mean	9.34	44.20	6.76	0.1846	0.1731	14.33	0.8977	1.34	0.7811	7.06

Abbreviations: CV, coefficient of variation; *R*_1_, baking loss (%); *R*_10_, overall acceptability; *R*_2_, L*_crust_; *R*_3_, b*_crust_; *R*_4_, springiness; *R*_5_, cohesiveness; *R*_6_, total dietary fiber content (g/100 g); *R*_7_, total phenolic content (mg QE/g); *R*_8_, antioxidant capacity measured by DPPH method (mg TEAC/g); *R*_9_, antioxidant capacity measured by ABTS method (mg TEAC/g).

**TABLE 3:** Model equations based on actual factors for the response variables.

Dependent variables	Model	Regression equation
Baking loss	Reduced quadratic model	*R*_1_ = 10.639 − 0.118*X*_2_ + 0.002*X*_2_^2^
L*_crust_	Linear model	*R*_2_ = 49.517 − 0.095*X*_1_ − 0.136*X*_2_
b*_crust_	Linear model	*R*_3_ = 11.131 − 0.053*X*_1_ − 0.132*X*_2_
Springiness	Reduced quadratic model	*R*_4_ = 0.263 − 0.003*X*_1_ − 0.003*X*_2_ + 0.00005*X*_1_^2^ + 0.00005*X*_2_^2^
Cohesiveness	Reduced quadratic model	*R*_5_ = 0.255 − 0.006*X*_2_ + 0.00009*X*_2_^2^
Dietary fiber content	Linear model	*R*_6_ = 0.075 + 0.306*X*_1_ + 0.326*X*_2_
TPC	Linear model	*R*_7_ = 0.232 + 0.014*X*_1_ + 0.014*X*_2_
Antioxidant capacity (DPPH method)	Linear model	*R*_8_ = 0.092 + 0.027*X*_1_ + 0.028*X*_2_
Antioxidant capacity (ABTS method)	2FI model	*R*_9_ = 0.106 + 0.007*X*_1_ + 0.012*X*_2_ + 0.0004*X*_1_ *X*_2_
Overall acceptability	Quadratic model	*R*_10_ = 6.731 + 0.025*X*_1_ + 0.018*X*_2_ − 0.000035*X*_1_ *X*_2_ − 0.0006*X*_1_^2^ − 0.0003*X*_2_^2^

*Note:* Terms are significant at *p* ≤ 0.05.

### 3.2 Effect of Variables on Physical Characteristics

**FIGURE 1:**
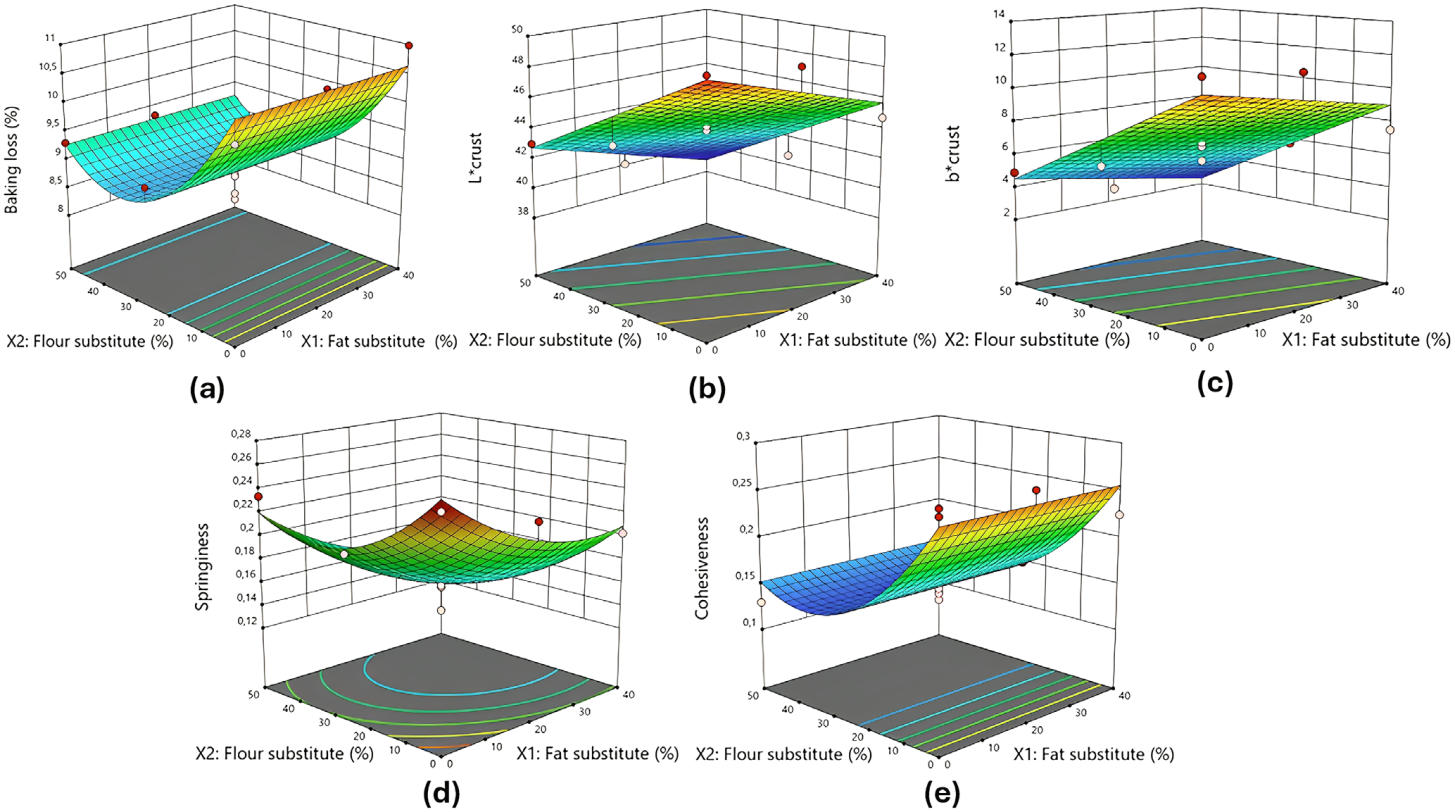
Response surface 3D graphs about the interaction effects of fat and rice flour substitute (%) on baking loss (a), L*_crust_ (b), b*_crust_ (c), springiness (d), and cohesiveness (e).

Baking loss in gluten‐free cake samples with different SCG substitutions varied between 8.29%–10.98% (Table 1). The independent variables affecting baking loss were evaluated with a reduced quadratic model, which was statistically significant (*p* < 0.05) and had a high coefficient of fit (*R* ^2^ = 0.861) (Tables 2 and 3). The lack of fit was not significant (*p* = 0.589), confirming the validity of the model (Table 2). In the model, the linear positive (*X*_2_) and second‐order negative effects of the flour substitution rate of SCG (*X*_2_^2^) had statistically significant effects (*p* < 0.05) (Tables 2 and 3). The response surface plot (Figure 1a) showed that as *X*_2_ increased, baking loss initially decreased but increased again above a certain threshold value (*X*_1_: 0%, *X*_2_: 25%). This result can be associated with the water‐holding capacity of SCGs (Ballesteros et al. 2014). The high moisture‐holding capacity fibers and components found in SCG can reduce water loss by retaining moisture, but this can reverse as the structure becomes unstable. Similar trends were observed in the study by Aydogdu et al. (2018), where oat, pea, and apple fiber were used instead of wheat flour in cake products. The study stated that the fiber‐substituted cakes had lower baking loss values than the control cake. Also, in another study using watermelon rind powder as a rice flour substitute in gluten‐free cakes, it was determined that baking loss decreased statistically as the watermelon rind powder substitution rate increased (*p* < 0.05) (Çelik 2021).

In gluten‐free cake samples with different SCG substitutions, L*_crust_ values ranged between 40.09–49.75, whereas b*_crust_ values changed between 2.77 and 12.08 (Table 1). The effects of independent variables on the L*_crust_ and b*_crust_ values of SCG‐substituted gluten‐free cakes were explained by linear models (Table 3). Regression analysis revealed a high coefficient of determination (*R* ^2^ = 0.871) and a non‐significant lack of fit (*p* = 0.081) for L*_crust_ values and a high coefficient of determination (*R* ^2^ = 0.858) and a non‐significant lack of fit (*p* = 0.102) for b*_crust_ values (Table 2). The linear negative effects of fat substitution rate (*X*_1_) and flour substitution rate (*X*_2_) of SCG on the L*_crust_ and b*_crust_ values of the gluten‐free cakes were statistically significant (*p* < 0.05) (Tables 2 and 3). The response surface plot (Figure 1b,c) indicated that L*_crust_ and b*_crust_ values decreased linearly with the increase of *X*_1_ and *X*_2_. Because the cake samples become darker with increasing SCG content, and the b*_crust_ value in gluten‐free cake color exhibits bluish tones (Dadalı 2023). This change can be associated with the brown pigment content of SCGs and the Maillard reactions occurring during the cooking process (Freitas et al. 2023). A similar situation was observed in a study by Benincá et al. (2023) when different amounts of SCGs were added to the muffin. In the study, a decrease in L* values was observed as the SCG content increased, and it was emphasized that this was due to the formation of melanoid associated with the addition of SCG during roasting of coffee grounds. Also, in the study conducted by Ali et al. (2018), a similar trend was observed when SCG was added to biscuit products at different rates (2%, 4%, 6%).

The springiness values of gluten‐free cake samples with different SCG substitutions ranged from 0.13 to 0.25, and their cohesiveness values ranged from 0.12 to 0.27 (Table 1). Reduced quadratic models were used to elucidate the effects of independent variables on springiness and cohesiveness values of gluten‐free cakes, and these models were found to be statistically significant (*p* < 0.05). According to the regression analysis results, high coefficients of determination (*R* ^2^ = 0.899 and 0.883) and non‐significant lack of fit values (*p* = 0.468 and *p* = 0.193) were detected for springiness and cohesiveness, respectively (Table 2). The negative linear (*X*_2_) and positive quadratic effects (*X*_2_^2^) of flour substitution rate of SCG displayed significant effects on both springiness and cohesiveness values of gluten‐free cakes, whereas the negative linear (*X*_1_) and positive quadratic effects (*X*_1_^2^) of fat substitution rate of SCG were statistically significant only on the springiness values (*p* < 0.05) (Tables 2 and 3). As seen in the response surface plot (Figure 1d), as *X*_1_ and *X*_2_ increased, springiness first decreased but then increased again above a certain threshold value (for fat substitution = *X*_1_: 20%, *X*_2_: 0% and for flour substitution = *X*_1_: 0%, *X*_2_: 25%). This nonlinear behavior is consistent with the *X*_1_^2^ and *X*_2_^2^ terms in the quadratic regression equation (Table 3). Due to its fiber‐rich structure and high water‐holding capacity, SCG weakens structural integrity at low substitution rates and softens the cake structure at high substitution rates because of increased water retention (Sudha et al. 2007; Ballesteros et al. 2014; Ahanchi et al. 2024). Furthermore, low springiness values in gluten‐free cake formulations can be attributed to the absence of an elastic network structure formed by gluten and the hardening effect of SCG's high fiber content on the structure. Thus, the gluten‐free cakes had limited elastic recovery capacity after compression. Gluten‐free cakes containing SCG exhibited a more brittle and less recoverable structure.

The cohesiveness value of gluten‐free cakes decreased quadratically as *X*_2_ increased, as depicted in the response surface plot (Figure 1e). Structural integrity decreased, particularly with increasing the use of SCG as a flour substitute. The decrease in structural integrity with increasing SCG substitution rate in the formulation may be due to SCG's inability to create a binding effect in the dough matrix. This is a consequence of its high fiber content and particulate structure (Gularte et al. 2012; Martins et al. 2017). Similar results have been observed in previous cake studies (Gómez et al. 2010; Hussein et al. 2019), where the springiness and cohesiveness of SCG‐substituted cakes reduced compared to the control cake.

### 3.3 Effect of Variables on Chemical Characteristics

**FIGURE 2:**
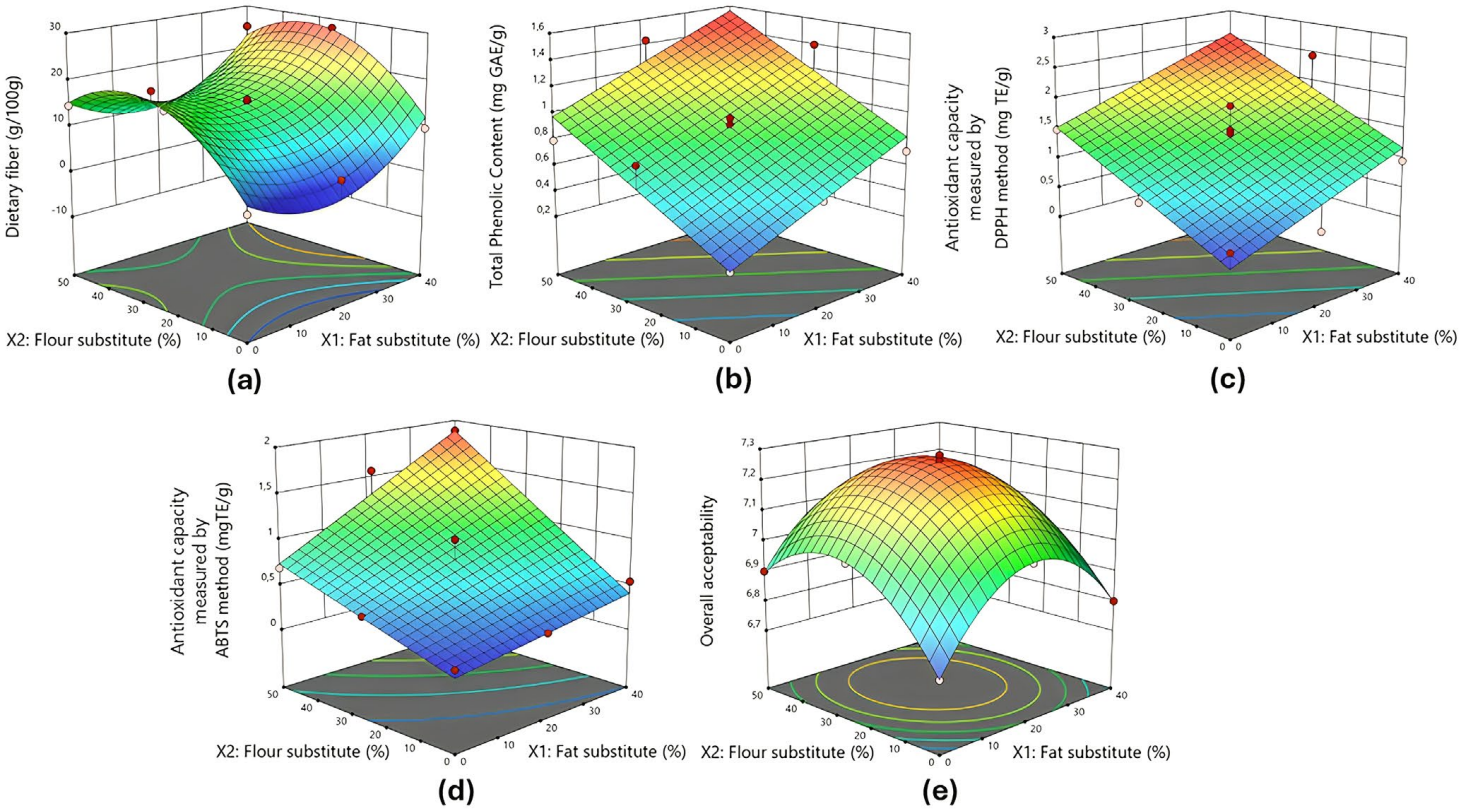
Response surface 3D graphs about the interaction effects of fat and rice flour substitute (%) on dietary fiber (a), TPC (b), antioxidant capacity measured by DPPH method (c), antioxidant capacity measured by ABTS method (d), and overall acceptability (e).

The dietary fiber content ranges of gluten‐free cake samples with different SCG substitutions were 2.3–27.9 g/100 g (Table 1). The ANOVA results of the linear model showing the effects of independent variables on the dietary fiber content of gluten‐free cakes are presented in Table 2. The model was statistically significant (*p* < 0.05), had a high coefficient of fit (*R* ^2^ = 0.921), and the lack of fit was not significant (*p* = 0.085) (Table 2). The linear terms of all independent variables are statistically significant (*p* < 0.05) (Tables 2 and 3). The linear effects of the fat substitution rate (*X*_1_) and flour substitution rate (*X*_2_) had a positive effect on the model (Tables 2 and 3). The response surface plot (Figure 2a) depicted that dietary fiber values followed a linear trend with the increase of *X*_1_ and *X*_2_. As *X*_1_ and *X*_2_ increased, the dietary fiber contents of gluten‐free cakes increased simultaneously. Since SCGs were a rich source of dietary fiber, their use instead of flour and fat in the formulation increased the fiber content of the final product (Martinez‐Saez et al. 2017; Koay et al. 2023). Similar trends were observed in the study conducted by Campos‐Vega et al. (2020), where biscuits enriched with different types of SCG‐based ingredients were developed. The incorporation of different types of SCG‐based ingredients into the biscuit formulation significantly increased the total dietary fiber content of the samples (8.4%–11.8%) (*p* < 0.05). In the study by Trà et al. (2021), as the ratio of SCG to flour weight in the cookies increased from 0 to 0.25, the total dietary fiber content of the samples increased by 1.7%–10.7% on a dry basis.

In gluten‐free cake samples with different SCG substitutions, TPC values were in the range of 0.22–1.46 mg QE/g. Antioxidant capacities measured by the DPPH method changed from 0.19 to 2.43 mg TEAC/g, whereas those measured by the ABTS method varied from 0.19 to 1.87 mg TEAC/g (Table 1). The independent variables affecting TPC and DPPH were assessed using statistically significant linear models (*p* < 0.05) (Table 3). The *R* ^2^ values were found to be 0.878 (TPC) and 0.813 (DPPH) and had a high coefficient of fit (Table 2). A significant two‐factor interaction (2FI) model was used to elucidate the effects of independent variables on antioxidant capacity measured by the ABTS method (*p* < 0.05), and the model had a high coefficient of determination (*R* ^2^ = 0.901) (Tables 2 and 3). The lack of fit in all methods was not significant (*p* = 0.101 for TPC, *p* = 0.397 for antioxidant capacity measured by the DPPH method, and *p* = 0.666 for antioxidant capacity measured by the ABTS method), confirming the adequacy of the models (Table 2). The positive linear terms of fat substitution rate (*X*_1_) and flour substitution rate (*X*_2_) of SCG had significant effects on the TPC and antioxidant capacity values of gluten‐free cakes, whereas the interaction term of fat substitution rate and flour substitution rate (*X*_1_ *X*_2_) showed a significant positive effect only on the antioxidant capacity values of gluten‐free cakes measured by the ABTS method (*p* < 0.05) (Tables 2 and 3). This suggested that the interactive effect of *X*_1_ and *X*_2_ might provide a synergistic contribution to increasing antioxidant capacity. As seen in the response surface plot (Figure 2b–d), the values of TPC and antioxidant capacity increased linearly with the increase in the usage rates of SCG as fat and flour substitute. These results demonstrated that SCGs were a rich source of polyphenols with antioxidant properties, and these compounds could be successfully transferred into the gluten‐free cake formulation (Hussein et al. 2019; Franca and Oliveira 2022; Meerasri and Sothornvit 2022). Thus, a healthier gluten‐free cake with high polyphenol content and antioxidant capacity was obtained compared to the control. This trend regarding polyphenol content and antioxidant capacity was consistent with the study conducted by Koay et al. (2023), who revealed that adding SCGs to cookie samples produced similar functional results. In addition, the study performed by Severini et al. (2020) also reported that the values of TPC and antioxidant activity determined by the DPPH method increased as the SCG ratio substituted into muffin samples increased. Similarly, in the study of Castaldo et al. (2021), it was stated that antioxidant capacity increased significantly (*p* < 0.05) in biscuits formulated with SCG.

### 3.4 Effect of Variables on Sensory Characteristics

Overall acceptability values in gluten‐free cake samples with different SCG substitutions were determined to be between 6.73 and 7.28 (Table 1). The effects of the independent variables on the overall acceptability values were elucidated using a quadratic model that was statistically significant (*p* < 0.05), had a high coefficient of fit (*R* ^2^ = 0.997), and the lack of fit was not significant (*p* = 0.443) (Tables 2 and 3). The fat substitution rate (*X*_1_) and flour substitution rate (*X*_2_) of SCG had a positive effect on the overall acceptability value, whereas their interaction (*X*_1_ *X*_2_), and second‐order effects (*X*_1_^2^ and *X*_2_^2^) showed statistically significant negative effects (*p* < 0.05) (Tables 2 and 3). The response surface plot (Figure 2e) demonstrated that overall acceptability values exhibited a parabolic trend with the increase of *X*_1_ and *X*_2_. The increase in both *X*_1_ and *X*_2_ primarily affected the overall acceptability positively; however, this effect decreased beyond a certain level; model coefficients significantly confirmed these changes (Figure 2e and Table 3). Overall acceptability values indicate that the product was liked by consumers but not highly preferred. This may be due to consumers encountering an unfamiliar taste or texture in the cake samples. The highest values of overall acceptability were obtained in combinations of moderate fat and flour replacement. Similar results were obtained in a study where artichoke leaves were used instead of fat and wheat flour in cakes (Dadalı 2023).

### 3.5 Optimization of Gluten‐Free Cake Formulation With SCG Substitution and Validation of Predicted Model

In this study, multi‐response optimization and desirability function approach were utilized to simultaneously obtain the most appropriate levels of different quality parameters (i.e., physical properties, dietary fiber and total polyphenol contents, antioxidant capacity, and overall acceptability characteristics of SCG‐substituted gluten‐free cake). For this purpose, TFCs, antioxidant capacities determined by DPPH and ABTS methods, dietary fiber contents, springiness, cohesiveness, and overall acceptability values were maximized. The baking loss values were kept within a certain range, whereas the color parameters of L*_crust_ and b*_crust_ were adjusted to reach those of the control sample. As a result of the optimization performed in the Design Expert 13.0 program, it was determined that the optimum formulation was a gluten‐free cake formulation containing 36.75% SCG as a fat substitute and 12.38% SCG as a rice flour substitute. Gluten‐free cakes were produced in five replicates using the optimum formulation, and the experimentally measured response values were compared with the predicted data from the model. An independent sample *t*‐test was performed to determine whether the differences between the experimental values and the predicted values from the model were significant at the 95% significance level. It was determined that the experimental and predicted values were compatible and there was no statistically significant difference (*p* < 0.05) (Table 4). Percentage error values were between 0.85% and 13.51% and were at acceptable levels. Thus, it was experimentally verified that the models obtained through optimization were successful.

**TABLE 4:** The validation results for predicted and experimental values.

Response	Predicted value	Experimental value^a^	Difference	%Error^b^	Standard error of the mean	*p*
Baking loss (%)	9.45	9.37 ± 0.02	0.08	0.85	0.01	0.820
L*crust	44.31	43.87 ± 0.67	0.44	1.00	0.48	0.789
b*crust	7.54	8.06 ± 0.06	−0.52	6.45	0.04	0.737
Springiness	0.17	0.18 ± 0.01	−0.01	5.56	0.01	0.659
Cohesiveness	0.19	0.18 ± 0.01	0.01	5.56	0.01	0.674
Dietary fiber (g/100 g)	15.35	15.71 ± 0.81	−0.36	2.29	0.57	0.911
TPC (mg QE/g)	0.96	1.11 ± 0.08	−0.15	13.51	0.06	0.487
Antioxidant capacity (DPPH method) (mg TEAC/g)	1.44	1.59 ± 0.02	−0.15	9.43	0.01	0.747
Antioxidant capacity (ABTS method) (mg TEAC/g)	0.73	0.78 ± 0.01	−0.05	6.41	0.01	0.839
Overall acceptability	7.03	6.75 ± 0.21	0.28	4.15	0.15	0.312

^a^ Mean ± SD of five replicate measurements.

^b^ % Error = (|yexp − ypre|/yexp) × 100.

### 3.6 Characterization of Optimum Gluten‐Free Cake With SCG Substitution and Control Gluten‐Free Cake

The physical, chemical, and sensory properties of the control gluten‐free cake and the gluten‐free cake containing the optimum level of SCG are shown in Table 5. SCG substitution significantly affected the moisture content of the gluten‐free cake samples (*p* < 0.05). The moisture content of the gluten‐free cake containing the optimum level of SCG was lower than that of the control gluten‐free cake (*p* < 0.05). Similarly, a decrease in moisture content was observed in the cookie samples due to SCG substitution (Sharma et al. 2021; Oliveira Batista et al. 2023). Also, a positive correlation was obtained between moisture contents and baking loss values. The high insoluble fiber content of SCG reduced moisture content, resulting in less water evaporation during baking and a reduction in baking loss (Ballesteros et al. 2014; Trà et al. 2021). Similarly, in a study where different amounts of SCG were added to bread, the bread containing the highest amount of SCG (10%) had a lower baking loss value (9.29%) (Daniel 2018).

**TABLE 5:** Physical, chemical and sensory properties of control gluten‐free cake and optimum gluten‐free cake with SCG substitution.

Analyses	Control gluten‐free cake	Gluten‐free cake containing the optimum level of SCG
Moisture (%)	25.47 ± 0.73^b^	21.13 ± 0.95^a^
Ash (%)	0.87 ± 0.03^a^	1.02 ± 0.12^a^
Fat (%)	11.65 ± 0.24^a^	16.40 ± 0.61^b^
Protein (%)	5.21 ± 0.04^a^	6.41 ± 0.13^b^
Carbohydrate (%)	56.76 ± 0.82^a^	55.10 ± 0.17^a^
Energy (kcal/100 g)	227.67 ± 4.24^a^	257.47 ± 3.70^b^
TPC (mg GAE/g)	0.23 ± 0.00^a^	1.11 ± 0.08^b^
Antioxidant capacity (DPPH method) (mg TEAC/g)	0.34 ± 0.01^a^	1.59 ± 0.02^b^
Antioxidant capacity (ABTS method) (mg TEAC/g)	0.19 ± 0.01^a^	0.78 ± 0.01^b^
Dietary fiber (g/100 g)	2.30 ± 0.16^a^	15.71 ± 0.81^b^
Baking loss (%)	10.27 ± 0.10^b^	9.37 ± 0.02^a^
Specific volume (cm^3^/g)	1.80 ± 0.02^b^	1.56 ± 0.00^a^
L*_crust_	49.75 ± 0.24^b^	43.87 ± 0.67^a^
b*_crust_	12.08 ± 0.49^b^	8.06 ± 0.06^a^
Hardness (N)	20.50 ± 0.98^a^	28.56 ± 2.12^b^
Springiness	0.25 ± 0.02^b^	0.18 ± 0.01^a^
Cohesiveness	0.27 ± 0.00^b^	0.18 ± 0.01^a^
Overall acceptability	6.73 ± 0.39^a^	6.75 ± 0.21^a^

*Note:* Mean ± SD of three replicate measurements. Each line is evaluated within itself, and different letters represent statistically significant differences (*p* < 0.05).

No statistically significant difference was found between the ash contents of the control gluten‐free cake and the gluten‐free cake containing the optimum level of SCG (*p* > 0.05). The results regarding their ash contents were consistent with a study conducted by Hussein et al. (2019). However, SCG substitution significantly affected the fat and protein contents of the gluten‐free cake samples (*p* < 0.05). The fat and protein contents of the gluten‐free cake containing the optimum level of SCG were observed to increase with the addition of SCG. Likewise, there was an increase in the fat and protein content of the cookie samples due to the SCG substitution (Vázquez‐Sánchez et al. 2018; Azuan et al. 2020). This is a result of the high fat and protein content of SCG (Bijla et al. 2022).

There was no statistically significant difference between the carbohydrate contents of the control gluten‐free cake and the gluten‐free cake containing the optimum level of SCG (*p* > 0.05). The results were in line with a study by Oliveira Batista et al. (2023). The energy content of the gluten‐free cake containing the optimum SCG level was statistically higher compared to the control sample (*p* < 0.05). This finding agreed with a previous study by Vázquez‐Sánchez et al. (2018), who determined a significant difference (*p* < 0.05) in energy content between the control sample and the biscuit sample substituted with antioxidant dietary fiber from SCG.

Substitution of SCG significantly increased the TPC content and antioxidant capacity of the gluten‐free cake sample (*p* < 0.05). This is because of the high TPC content and antioxidant capacity of light and medium roast coffees (Benincá et al. 2023). Meerasri and Sothornvit (2022) reported that replacing butter with fat derived from SCGs in cookie formulations increased the TPC values of cookies. SCG is reported to be a natural antioxidant source (Castaldo et al. 2021). Similarly, the antioxidant values of cookie samples increased with the addition of SCG to the formulation (Azuan et al. 2020; Sharma et al. 2021). Studies generally emphasized a positive correlation between measured TPC value and antioxidant capacity (Kiss et al. 2025; Walasek‐Janusz et al. 2025). Furthermore, the results indicated that SCG could be considered not only as a replacement ingredient but also as a functional additive (Montemurro et al. 2024).

The SCG substitution increased the dietary fiber content of the gluten‐free cake containing the optimum level of SCG (15.71 g/100 g) by approximately 7‐fold compared to the control gluten‐free cake (2.3 g/100 g). This is a consequence of SCG being a very rich source of dietary fiber (Castaldo et al. 2021; Bijla et al. 2022). According to the European Union's nutrition claims regulation, cookies containing optimum levels of SCG are considered a source of dietary fiber (dietary fiber ≥ 3 g/100 g) (European Commission 2008). Therefore, the gluten‐free cake containing the optimum level of SCG can be said to be a source of dietary fiber. The addition of SCG to various samples resulted in increased fiber content compared to the control samples in previous research (*p* < 0.05) (Sharma et al. 2021; Trà et al. 2021; Oliveira Batista et al. 2023).

The specific volume of the gluten‐free cake containing the optimum level of SCG was found to be lower than the volume of the control gluten‐free cake (*p* < 0.05). The results showed that the presence of SCG in optimum cake samples reduced gas retention due to the high insoluble dietary fiber content (Aghajanzadeh et al. 2024). Similarly, a study by Ateş and Elmacı (2019) observed that the specific volumes of cakes containing coffee silverskin decreased compared to control samples. The L*_crust_ and b*_crust_ values of the gluten‐free cake containing the optimum level of SCG were found to be lower than the values of the control gluten‐free cake (*p* < 0.05). The results showed that the color of the cake crust became darker, less yellow, and paler. The darkening observed in the color of cakes with the addition of SCG could be explained by the contribution of melanoidins and Maillard reaction products naturally present in SCG, and these compounds were reported to cause a decrease in L* and b* values (Iriondo‐DeHond et al. 2021; Ahanchi et al. 2024). Similarly, a study by Papageorgiou et al. (2024) observed that the color values (L* and a*) of cookies containing SCG decreased compared to control samples.

Substituting SCG at the optimum concentration in a gluten‐free cake caused a decrease in springiness and cohesiveness values and an increase in hardness value (*p* < 0.05). The increase in hardness resulted in a harder cake texture. The decrease in elasticity led to a decrease in the elasticity of the gluten‐free cake, whereas the decrease in cohesiveness made the gluten‐free cakes more prone to breakage and crumbling (Ahanchi et al. 2024). The result about the hardness value agrees with the findings of Gularte et al. (2012) when up to 20% levels of soluble (inulin and guar gum) and insoluble (oat fiber) fibers were added to gluten‐free cakes. Rwubatse et al. (2021) stated that there was a decrease in elasticity and cohesion values in breads containing SCG. In the gluten‐free cake structure, SCG is thought to create a more compact, breakable, and hard structure by reducing water distribution and gas retention (Aydogdu et al. 2018).

According to sensory evaluation results, the gluten‐free cake and the gluten‐free cake containing the optimum level of SCG were statistically similarly appreciated by panelists (*p* > 0.05). The caffeine content of SCGs is a key consideration, especially for sensitive consumer groups. The caffeine content of SCGs is quite low, at approximately 14.5 μg/g, due to the substantial transfer of caffeine into the beverage during brewing (Bomfim et al. 2022; Bevilacqua et al. 2023). Given the low initial caffeine content of SCGs and their dilution effect on the cake matrix, the addition of SCGs did not exert a negative effect on the final product. The absence of negative feedback regarding bitter taste from panelists in sensory analyses also supports this conclusion. Even in gluten‐free cakes with the highest SCG content, no bitter taste was perceived. Thus, the new gluten‐free cake, formulated by adding SCG as fat and rice flour substitute to the gluten‐free cake, appeared to be a preferred gluten‐free alternative by consumers.

## 4 Conclusion

In recent years, the depletion of natural resources coupled with the increasing global population has increased the need for alternative food sources with functional properties and health benefits. SCG is a food waste product that stands out with its rich dietary fiber content and potential for environmental sustainability. In this study, SCG was used as a rice flour and fat substitute in a gluten‐free cake, and the optimum gluten‐free cake formulation was determined to contain 12.38% rice flour and 36.75% fat substitute. The use of SCG at optimum levels as rice flour and fat substitute in gluten‐free cake formulation increased the protein, fat, dietary fiber, TPC, and antioxidant capacity contents, energy, and hardness values, and overall acceptability of the cake. One of the most striking findings of this study was the significant increase observed in dietary fiber content. The content of dietary fiber, determined as 2.3 g/100 g in the control gluten‐free cake, reached 15.71 g/100 g in the gluten‐free cake containing the optimum level of SCGs, showing approximately a 7‐fold increase. This significant increase was due to the high dietary fiber content of SCG and revealed a substantial improvement in the nutritional value of the gluten‐free cakes. Substituting SCG, a food waste, in the gluten‐free cake can contribute to environmental sustainability and enhance the nutritional value of the cake. This offers a functional snack for individuals looking for healthier food alternatives and preferring a gluten‐free diet.

## Author Contributions

**Emine Nakilcioğlu:** resources, data curation, formal analysis, writing – review and editing, writing – original draft, investigation, conceptualization, methodology, validation, visualization. **Gizem Tiryaki:** conceptualization, investigation, funding acquisition, writing – original draft, methodology, validation, visualization, writing – review and editing, software, project administration, data curation, supervision, resources.

## Funding

This work was supported by Ege University Scientific Research Projects Coordination Office (Project No: 32836).

## Conflicts of Interest

The authors declare no conflicts of interest.

## Data Availability

The data that support the findings of this study are available from the corresponding author upon reasonable request.
